# DTM-Aided Adaptive EPF Navigation Application in Railways

**DOI:** 10.3390/s18113860

**Published:** 2018-11-09

**Authors:** Chengming Jin, Baigen Cai, Jian Wang, Allison Kealy

**Affiliations:** 1 School of Electronic and Information Engineering, Beijing Jiaotong University, Beijing 100044, China; wangj@bjtu.edu.cn; 2 School of Computing, Beijing Jiaotong University, Beijing 100044, China; bgcai@bjtu.edu.cn; 3 State Key Laboratory of Railway Traffic Control & Safety, Beijing Jiaotong University, Beijing 100044, China; 4 Beijing Engineering Research Center of EMC and GNSS Technology for Rail Transportation, Beijing 100044, China; 5 School of Geospatial Science, Royal Melbourne Institute of Technology, Melbourne VIC 3001, Australia; allison.kealy@rmit.edu.au

**Keywords:** adaptive filtering, extended Kalman particle filter, digital track map, train navigation application

## Abstract

The diverse operating environments change GNSS measurement noise covariance in real time, and different GNSS techniques hold different measurement noise covariance as well. Mismodelling the covariance causes undependable filtering results and even degenerates the GNSS/INS Particle Filter (PF) process, due to the fact that INS error-state noise covariance is much smaller than that of GNSS measurement noise. It also makes the majority of existing methods for adaptively adjusting filter parameters incapable of performing well. In this paper, a feasible Digital Track Map-aided (DTM-aided) adaptive extended Kalman particle filter method is introduced in GNSS/INS integration in order to adjust GNSS measurement noise covariance in real time, and the GNSS down-direction offset is also estimated along with every sampling period through making full use of DTM information. The proposed approach is successfully examined in a railway environment, and the on-site experimental results reveal that the adaptive approach holds better positioning performance in comparison to the methods without adaptive adjustment. Improvements of 62.4% and 14.9% in positioning accuracy are obtained in contrast to Standard Point Positioning (SPP) and Precise Point Positioning (PPP), respectively. The proposed adaptive method takes advantage of DTM information and is able to automatically adapt to complex railway environments and different GNSS techniques.

## 1. Introduction

The Global Navigation Satellite System (GNSS) has been broadly utilized in aviation for decades. Subject to RAMS (Reliability, Availability, Maintainability and Safety) requirements, as well as economic reasons of safety-critical applications in railways, GNSS has not been popularly accepted by rail authorities. Due to its global capability of high accuracy and availability, GNSS, as a localization sensor, nevertheless plays a strategic role in the train control system of the next generation, e.g., the European Train Control System Level 3 (ETCS-3), the Chinese Train Control System (CTCS)and Positive Train Control (PTC) [1,2,3], and some pioneer lines have been deployed worldwide to testify the performance in actual train operating environments [4].

Standalone GNSS techniques, such as multi-constellation [5], carrier-phase-based GNSS [6,7] and tracking loop design [8,9], improve positioning accuracy and availability. GNSS generally integrates with other sensors, for instance Inertial Measurement Units (IMUs) [10,11], composing GNSS/Inertial Navigation System (INS) integration architectures, to better adapt to some GNSS restricted environments, for instance urban canyons and tunnels. The drawbacks and benefits of INS and GNSS are complementary. INS operates continuously, prevents hardware faults and provides high-bandwidth output; however, errors accumulate along with time; meanwhile, GNSS provides a long-term position within an absolute accuracy of a few meters [12]. There are two methods of solving the GNSS/INS nonlinear issue in the system or measurement model. The first option is to adopt linearized algorithms, e.g., the Extended Kalman Filter (EKF) [13,14,15], an improved Kalman Filter [16,17,18] optimal for linear systems where process noise and measurement noise are characterized by white noise, which is the most mature one, since it fully takes advantage of small angle approximation applied to INS error-state propagation and linearizes the measurement equation. The alternative methods are nonlinear filtering algorithms, e.g., Particle Filter (PF) [19,20].

This paper focuses on the PF-based algorithm due to its better capability of modelling nonlinear/non-Gaussian system propagation. Classical particle filter algorithms are prone to degeneration because of small state noise in INS error-state propagation. Some improved PFs have been investigated to tackle this issue, among which GNSS/INS Extended Kalman Particle Filter (EPF) [21] and Unscented Kalman Particle Filter (UPF) [22,23] are proven effective with great success. Another fact is GNSS measurement noise covariance is subject to either diverse environmental conditions or variable GNSS positioning techniques, e.g., multi-constellation, Differential GNSS (DGNSS), Real-Time Kinematic (RTK) and Precise Point Positioning (PPP) [24]. Therefore, an adaptive PF is requisite for applying PF in a real operating environment. The augmented PF [25] had been proposed; however, it deals with an uncertain dynamic system model and is unable to estimate and dynamically adjust measurement noise covariance. Meanwhile, some Kullback-Leibler Divergence (KLD)-based algorithms [26,27,28] were proposed for the adaptive measurement model. The KLD-based approach requires that the functional form of the measurement model is known, but not the value of its parameters. In addition, it is sensitive to true measurements since minimizing KLD shifts the probability distribution of the system process model to the measurement model [28]. That is, the noisy measurements will cause the failure of filtering, which holds the truth in GNSS/INS PF, because GNSS measurements are much noisier in comparison to INS error-state prediction. Other adaptive PF approaches [29,30,31] emphasize optimized particle set sizes and are inappropriate for adjusting measurement noise covariance.

Alternatively, extra information introduced to filters is able to aid the filtering process. In railways, the trains are constantly constricted on tracks, and the Digital Track Map (DTM) precisely incorporates track information, i.e., 3D position, even attitude. The map provides environmental information to deter particles from being in impossible real physical space, like crossing a wall [32]. The work in [33] used the particle filter to estimate a topological position directly in the track map. The work in [34] introduced the track constraint to create eligible particles. None of these methods showed any improved performance if a misleading GNSS measurement noise covariance were adopted. The proposed adaptive EPF in this paper is constructed on DTM, which provides train position information to adjust GNSS measurement noise covariance over the period of PF, so as to model the GNSS measurement noise adaptively.

The rest of the paper is organized as follows, GNSS/INS PF and EPF algorithms are firstly introduced in Section 2. Then, combined with EKF in Section 2, the theory and implementation of DTM-aided adaptive GNSS/INS EPF are described in detail in Section 3. Later on, an application in a real train positioning scenario is presented in Section 4, and the on-site experimental results compare the performance of positioning with conventional EPF and EKF algorithms, which do not adjust GNSS measurement noise covariance adaptively. Finally, Section 5 concludes this paper and discusses some possible application challenges.

## 2. GNSS/INS Extended Kalman Particle Filter

The GNSS/INS EKF and an improved Sampling Importance Resampling (SIR) filter are described in this section. The EKF will be used in Section 3 to assist the SIR filter to estimate and update GNSS measurement noise covariance matrix; thus, a better described density can be obtained to guide the importance sampling of the SIR filter, which is described in Section 3 as well.

### 2.1. GNSS/INS Extended Kalman Filter

#### 2.1.1. System Model

The most common GNSS/INS fusion method is the loosely-coupled integration of 15 error-state vector $x_{k}$ [10], which is given by:

(1) $$x_{k}=\left(\begin{array}{c} \delta \psi_{eb}^{e}\\ \delta v_{eb}^{e}\\ \delta r_{eb}^{e}\\ b_{a}\\ b_{g} \end{array}\right)$$

where $\delta \psi_{eb}^{e}$ denotes angular-rate error, $\delta v_{eb}^{e}$ represents velocity error, $\delta r_{eb}^{e}$ is position error and $b_{a}$ and $b_{g}$ denote accelerometer biases and gyro biases, respectively. The error-state vector-based system continuous model is thus:

(2) $$\left(\begin{array}{c} \delta {\dot{\psi }}_{eb}^{e}\\ \delta {\dot{v}}_{eb}^{e}\\ \delta {\dot{r}}_{eb}^{e}\\ {\dot{b}}_{a}\\ {\dot{b}}_{g} \end{array}\right)=\left(\begin{array}{ccccc} -\Omega_{ie}^{e} & 0_{3} & 0_{3} & 0_{3} & {\hat{C}}_{b}^{e}\\ F_{21}^{e} & -2\Omega_{ie}^{e} & F_{23}^{e} & {\hat{C}}_{b}^{e} & 0_{3}\\ 0_{3} & I_{3} & 0_{3} & 0_{3} & 0_{3}\\ 0_{3} & 0_{3} & 0_{3} & 0_{3} & 0_{3}\\ 0_{3} & 0_{3} & 0_{3} & 0_{3} & 0_{3} \end{array}\right)\left(\begin{array}{c} \delta \psi_{eb}^{e}\\ \delta v_{eb}^{e}\\ \delta r_{eb}^{e}\\ b_{a}\\ b_{g} \end{array}\right)+Gw_{s}=F_{k-1}x_{k-1}+Gw_{s}$$

(3) $$F_{21}^{e}=\left[-\left({\hat{C}}_{b}^{e}{\hat{f}}_{ib}^{b}\right)\wedge \right],F_{23}^{e}=\frac{2g0(\hat{L_{b}})}{r_{eS}^{e}\left(\hat{L_{b}}\right)}\frac{{\hat{r}}_{eb}^{e}}{|{\hat{r}}_{eb}^{e}{|}^{2}}{\left({\hat{r}}_{eb}^{e}\right)}^{T}$$

where $\Omega_{ie}^{e}$ is the skew-symmetric matrix of the angular rate, ${\hat{C}}_{\beta }^{\alpha }$ denotes the coordinate transformation matrix, which transforms a vector from the β coordinate frame to the α coordinate frame, g0 is the surface gravity, ${\hat{r}}_{eb}^{e}$ denotes an estimate of $r_{eb}^{e}$, which is the INS Cartesian position of the origin of Frame b with respect to the origin of Frame e, resolved about the axes of Frame e. $r_{eS}^{e}$ is the distance of a point *S* with respect to the Earth’s centre. ${\hat{f}}_{ib}^{b}$ is the IMU measurement specific force along the body-frame resolving axes. $\hat{L_{b}}$ denotes INS latitude estimate. G and $w_{s}$ denote the system noise distribution matrix and the system noise vector, respectively.

The system state vector transition matrix is given by:

(4) $$\Phi_{k-1}\approx I+F_{k-1}\tau_{s}$$

where $\tau_{s}$ denotes the Kalman Filter interval, not the IMU output interval.

#### 2.1.2. Measurement Model

GNSS/INS integration uses GNSS position $r_{ebG}^{e}$ and velocity $v_{eb}^{e}$ measurements, thus:

(5) $$\delta z_{k}=\left(\begin{array}{c} r_{ebG}^{e}-r_{eb}^{e}\\ v_{ebG}^{e}-v_{eb}^{e} \end{array}\right)$$

and measurement matrix $H_{k}$ is given by:

(6) $$H_{k}=\left(\begin{array}{ccccc} 0_{3} & 0_{3} & -I_{3} & 0_{3} & 0_{3}\\ 0_{3} & -I_{3} & 0_{3} & 0_{3} & 0_{3} \end{array}\right)$$

#### 2.1.3. Extended Kalman Filter Algorithm

The EKF algorithm comprises the following steps [12]:

(a) Calculate the transition matrix, $\Phi_{k-1}$;
(b) Calculate the system noise covariance matrix, $Q_{k-1}$;
(c) Propagate the state vector estimate from ${\hat{x}}_{k-1}^{+}$ to ${\hat{x}}_{k-1}^{-}$:
  (7) $${\hat{x}}_{k-1}^{-}=\Phi_{k-1}{\hat{x}}_{k-1}^{+}$$
(d) Propagate the error covariance matrix from $P_{k-1}^{+}$ to $P_{k}^{-}$:
  (8) $$P_{k}^{-}=\Phi_{k-1}P_{k-1}^{+}\Phi_{k-1}^{⊤}+Q_{k-1}$$
(e) Calculate the measurement matrix $H_{k}$;
(f) Calculate the measurement noise covariance matrix $R_{k}$;
(g) Calculate the Kalman gain matrix $K_{k}$:
  (9) $$K_{k}=P_{k}^{-}H_{k}^{⊤}{\left(H_{k}P_{k}^{-}H_{k}^{⊤}+R_{k}\right)}^{-1}$$
(h) Calculate the innovation $\delta z_{k}$;
(i) Update the state vector estimate from ${\hat{x}}_{k}^{-}$ to ${\hat{x}}_{k}^{+}$:
  (10) $${\hat{x}}_{k}^{+}={\hat{x}}_{k}^{-}+K_{k}\delta z_{k}$$
(j) Update the error covariance matrix from $P_{k}^{-}$ to $P_{k}^{+}$:
  (11) $$P_{k}^{+}=\left(I-K_{k}H_{k}\right)P_{k}^{-}$$

### 2.2. Improved Sampling Importance Resampling Filter

The SIR filter, also known as the bootstrap filter [35], along with the Sequential Importance Sampling (SIS) filter are basic particle filters. The SIS filter turns to an SIR filter by choosing the importance density to be the transitional prior; in addition, unlike the SIS filter conducting resampling if and only if a significant degeneracy is observed, SIR performs the resampling step in everyfiltering recursive cycle.

Firstly, let us introduce $X_{k}=\left\{x_{j},j=0,...,k\right\}$, which represents the system state sequence up to time *k*, $X_{j}^{i}=\left\{x_{j}^{i},j=0,...,k,i=1,...,N\right\}$, which is the sequence of *N* sampling points at time *j*. Suppose the multidimensional integral $I(X_{k})$ is given by:

(12) $$I\left(X_{k}\right)=\int X_{k}\left(x\right)dx$$

where $x\in R^{n_{x}}$. Based on Monte Carlo (MC) integration, it is possible to draw N≫1 sample sequence $X_{k}^{i}$; therefore, the MC estimate of integral $I(X_{k})$ is the sample mean:

(13) $$I_{N}\left(X_{k}\right)=\frac{1}{N}\sum_{i=1}^{N}x_{k}^{i}$$

where $X_{k}\left(x\right)=x_{k}\cdot \pi \left(x_{k}\right)$ and $\pi (x_{k})$ is a probability density function. The law of large numbers reveals that $I_{N}\left(X_{k}\right)$ will converge to $I(X_{k})$. Unfortunately, it is usually impossible to sample system states $X_{k}^{i}$ properly, which means we cannot use Equation (13) to estimate system states at time *k*.

The discovery of importance resampling and resampling techniques paves the way for the success of PF. At an importance resampling stage, it supposes samples from a similar density $q(x_{k})$ can be drawn. Then, Equations (12) and (13) can be rewritten respectively as:

(14) $$I\left(X_{k}\right)=\int x_{k}\cdot \frac{\pi (x_{k})}{q(x_{k})}\cdot q\left(x_{k}\right)dx$$

(15) $$I_{N}\left(X_{k}\right)=\frac{1}{N}\sum_{i=1}^{N}x_{k}^{i}\tilde{\omega }\left(x_{k}^{i}\right)$$

where the importance weights are given by:

(16) $$\tilde{\omega }\left(x_{k}^{i}\right)=\frac{\pi (x_{k}^{i})}{q(x_{k}^{i})}$$

Furthermore, applying importance sampling in the Bayesian framework, thus $\pi (x_{k})$ becomes the posterior density $p(x_{k}|Z_{k})$, where $Z_{k}$ denotes measurements up to time *k*. Therefore, Equations (15) and (16) can be rewritten as [36,37]:

(17) $$p\left(x_{k}|Z_{k}\right)\approx \sum_{i=1}^{N}\omega_{k}^{i}\delta \left(x_{k}-x_{k}^{i}\right)$$

(18) $$\begin{array}{cc} w_{k}^{i} & \propto \frac{p(X_{k}^{i}|Z_{k})}{q(X_{k}^{i}|Z_{k})}\\ & =w_{k-1}^{i}\frac{p\left(z_{k}|x_{k}^{i}\right)p\left(x_{k}|x_{k-1}^{i}\right)}{q(x_{k}^{i}|x_{k-1}^{i},z_{k})} \end{array}$$

where $\omega_{k}^{i}$ denotes normalized weights, i.e., $\sum_{i}\omega_{k}^{i}=1$, and $X_{k}^{i}$ represents samples drawn from an importance density $q(X_{k}|Z_{k})$.

Ideally, the importance density should be $p(X_{k}|Z_{k})$; thus, applying $q(X_{k}|Z_{k})$ causes the sample degeneracy phenomenon due to the increase of the variance of importance weights [37]. A resampling is introduced to eliminate low importance weight samples by a random measure. It selects some high weight samples to replace low ones.

The most popular transitional prior is given by:

(19) $$\begin{array}{cc} q(x_{k}^{i}|x_{k-1}^{i},z_{k}) & =p(x_{k}|x_{k-1}^{i}) \end{array}$$

Substitution of Equation (19) into (18) yields:

(20) $$\omega_{k}^{i}\propto \omega_{k-1}^{i}p\left(z_{k}|x_{k}^{i}\right)$$

However, because the resampling is applied in every SIR filter recursive cycle, it reveals that all particles have equal weights; thus:

(21) $$\omega_{k}^{i}\propto p\left(z_{k}|x_{k}^{i}\right)$$

In the case of GNSS/INS fusion, if Equation (19) is applied, Equation (21) causes particles to degenerate rapidly. An improved method is to use EKF to approximate importance density $p(X_{k}|Z_{k})$. The key is that EKF provides mean (${\bar{X}}_{k}$) and variance ($\hat{P_{k}}$) to guide importance resampling:

(22) $$p\left(X_{k}|Z_{k}\right)\approx N\left({\bar{X}}_{k},\hat{P_{k}}\right)$$

## 3. DTM-Aided Adaptive GNSS/INS EPF

In this section, the proposed DTM-aided GNSS covariance matrix estimation approach is firstly introduced, which is the key of the adaptive EPF algorithm, and then is combined with GNSS/INS EKF and the SIR filter explained in Section 2. A detailed DTM-aided adaptive GNSS/INS EPF architecture will be finally described.

### 3.1. DTM-Aided GNSS Covariance Matrix Estimate

**Figure 1:**
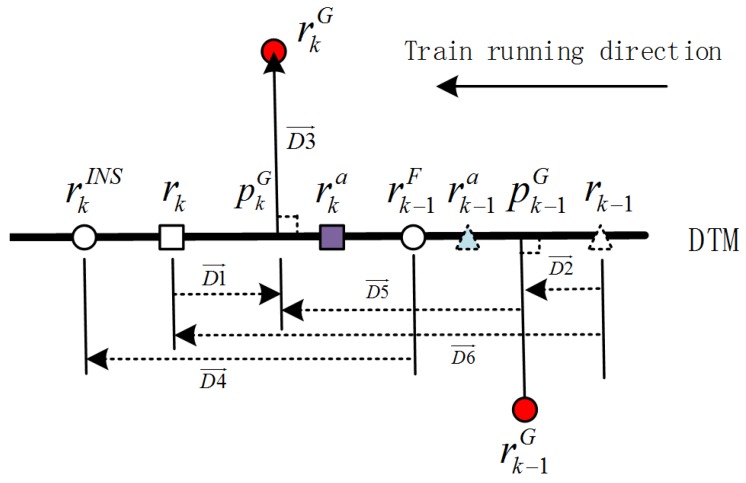
Plane of tracks.

The ultimate goal of different adaptive algorithms is to achieve the ability of estimating unknown parameters correctly and in real time. In the context of estimating the GNSS noise covariance matrix in a GNSS/INS integration, it usually involves adjusting the unknown parameter based on INS. In the rest of this paper, a high-definition DTM representing a central line of tracks is introduced to model the two parallel tracks. Thanks to the nature of the train navigation application, the train is constantly running on the tracks. That is, no matter what kind of GNSS technique is adopted and how the surrounding environment changes, the GNSS equipment mounted on the train is constantly located in a line described by DTM, if the height between GNSS and tracks is known and deducted previously. Moreover, assume the GNSS noise errors in Cartesian coordinates are independent and identically distributed. It is possible to construct an adaptive estimation algorithm, which is reported in Figure 1.

Figure 1 describes the plane of tracks, which mean the surface of two parallel track lines. The thick line denotes DTM, which includes a series of high-accuracy discrete Points Of Interest (POI). Define horizontal direction of DTM overlaps with that of plane of tracks, since DTM includes the centre line information, i.e., POI of tracks, while $r_{k}^{G}$, $r_{k-1}^{G}$ represent GNSS position measurements projected on the plane of tracks at time *k* and k−1, respectively. $r_{k-1}^{F}$ are the GNSS/INS fusion positioning results projected on the horizontal plane of DTM at time k−1. $r_{k}^{INS}$ indicates the distance the INS reckons along the DTM from the last fusion positioning point at time k−1 to time *k*. $r_{k}$ and $r_{k-1}$ are train real positions at time *k* and k−1, respectively. Due to the restriction of the tracks, the train is constantly running along the centre line of two parallel track lines. $p_{k}^{G}$ and $p_{k-1}^{G}$ are the cross-points of projecting $r_{k}^{G}$, $r_{k-1}^{G}$ respectively on DTM. $\vec{D1}$ represents a vector from $r_{k}$ to $p_{k}^{G}$; similar definitions of vectors $\vec{D2}$ to $\vec{D6}$ have been illustrated in Figure 1. Figure 1 also shows the direction that the train is running and the alternative positions of point $r_{k}$, $r_{k-1}$, which are denoted by $r_{k}^{a}$, $r_{k-1}^{a}$. Therefore, the uncertainty of the $r_{k}$, $r_{k-1}$ positions is described as:

(23) $$\left\{\begin{array}{c} \left(r_{k},r_{k-1}\right)\;|\;\vec{D2}-\vec{D1}=\vec{D6}-\vec{D5}\\ \left(r_{k},r_{k-1}^{a}\right)\;|\;\vec{D2}-\vec{D1}=\vec{D6}-\vec{D5}\\ \left(r_{k}^{a},r_{k-1}\right)\;|\;\vec{D2}-\vec{D1}=\vec{D6}-\vec{D5}\\ \left(r_{k}^{a},r_{k-1}^{a}\right)\;|\;\vec{D2}-\vec{D1}=\vec{D6}-\vec{D5} \end{array}\right.$$

It is further assumed that the distance INS reckons along DTM at time *k* is approximate to the GNSS measurement distance along DTM between time *k* and k−1. It gives:

(24) $$\vec{D4}\approx \vec{D6}$$

Substituting Equation (24) into (23) gives:

(25) $$\vec{D1}-\vec{D2}\approx \vec{D5}-\vec{D4}$$

If D1 and D2 follow the same zero-mean Gaussian distribution and are independent, $\sigma_{a}^{2}$ represents GNSS measurement noise variance along the DTM direction, $\sigma_{v}^{2}$ is the GNSS measurement noise variance orthogonal to DTM on the plane of tracks, $\sigma_{vv}^{2}$ denotes the GNSS measurement noise variance orthogonal to DTM in the direction vertical to the plane of tracks, point $p_{k}^{G,P}$ is the projection of the GNSS positioning point on the plane of tracks and $\vec{D8}$ denotes a vector from point $p_{k}^{G,P}$ to $r_{k}^{G}$. Therefore:

(26) $$\frac{\vec{D5}-\vec{D4}}{2}∼N\left(0,\sigma_{a}^{2}\right)$$

(27) $$\vec{D3}∼N\left(0,\sigma_{v}^{2}\right)$$

(28) $$\vec{D8}∼N\left(0,\sigma_{vv}^{2}\right)$$

Equations (26) and (27) combined with (28) provide a way of estimating GNSS measurement noise covariance matrix $R_{k}$. In comparison to other estimate algorithms, DTM considered as a sensor gives real GNSS measurement errors in two vertical directions. Meanwhile, because INS short-term error is relatively stable and smaller than that of GNSS, it is reasonable to estimate GNSS measurement noise along DTM by using INS information. A universal GNSS measurement noise variance $\sigma_{G}^{2}$ is given by:

(29) $$\left(\frac{\vec{D5}-\vec{D4}}{2}\right)+\vec{D3}+\vec{D8}∼N\left(0,\sigma_{G}^{2}\right)$$

### 3.2. DTM-Aided Adaptive GNSS/INS EPF Architecture

**Figure 2:**
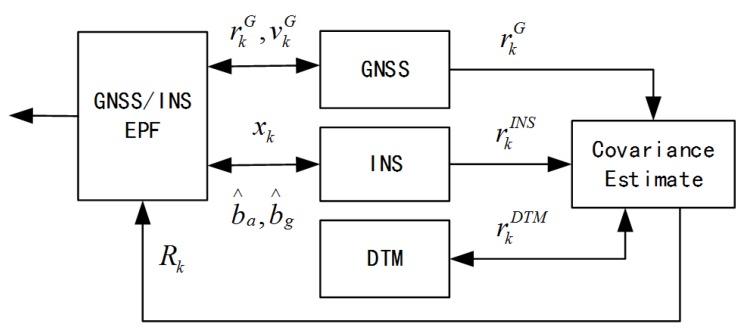
DTM-aided adaptive GNSS/INS Extended Kalman Particle Filter (EPF) architecture.

The proposed algorithm is based on the particle filter. In order to adjust GNSS measurement noise covariance, the DTM and covariance estimate modules are introduced. The DTM module provides position points $r_{k}^{DTM}$ and interpolates these points in support of calculating $p_{k}^{G}$. The covariance estimate module collects information from the GNSS, INS and DTM modules to estimate $R_{k}$ in real time. The GNSS/INS EPF module, as the main fusion module, accounts for the particle filter, e.g., importance resampling. The closed-loop architecture is illustrated in Figure 2.

In the closed-loop configuration, the average values of output particles in every recursive process, which represent estimated accelerometer, gyro, position, velocity and attitude errors, are used to correct IMU measurements, respectively INS. The IMU output frequency is usually higher than that of GNSS. *k* denotes the time when the GNSS measurement arrives. The proposed Algorithm 1 is given below.

**Algorithm 1:** Adaptive GNSS/INS EPF algorithm.

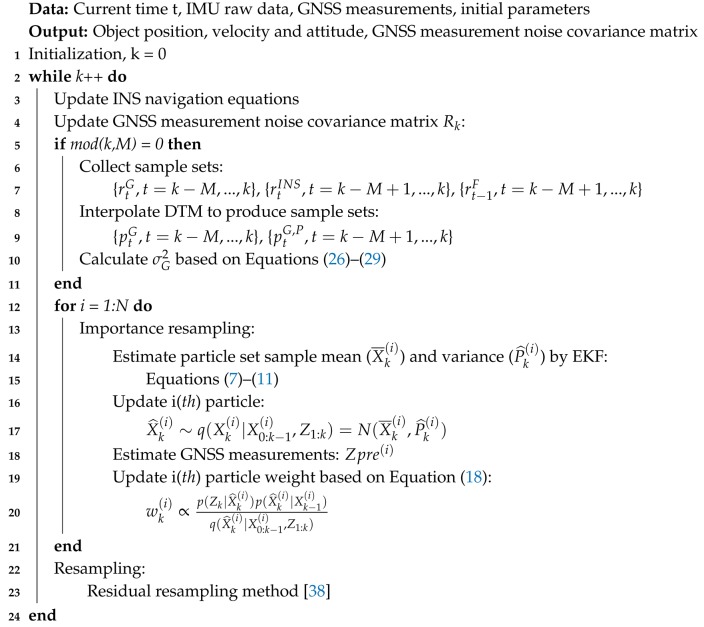



mod is modular operation; *M* denotes sample size; $Z_{k}$ represents GNSS measurements at time *k*. During time *k* and (k−M), the algorithm collects samples to estimate the GNSS measurement noise covariance matrix. A step of importance resampling is followed, which is the key of the improved EPF algorithm. It takes advantage of EKF and makes use of the EKF estimated particle mean and standard deviation to guide the importance resampling process. This means the algorithm uses EKF to help generate samples, which are approximate to the desired posterior density. randn(15) is a 15×15 diagonal matrix, in which the element is a random variable following the standard normal distribution,

(30) $${\hat{X}}_{k}^{\left(i\right)}={\bar{X}}_{k}^{\left(i\right)}+randn\left(15\right)\times {\hat{P}}_{k}^{\left(i\right)}$$

(31) $$Zpre^{\left(i\right)}=H_{k}{\hat{X}}_{k}^{\left(i\right)}$$

(32) $$p\left(Z_{k}|{\hat{X}}_{k}^{\left(i\right)}\right)=\frac{1}{{\left(2\pi \right)}^{3}{\left|R_{k}\right|}^{\frac{1}{2}}}exp(-\frac{1}{2}|R_{k}{|}^{-1}{\left(Z-Zpre^{\left(i\right)}\right)}^{2})$$

(33) $$p\left({\hat{X}}_{k}^{\left(i\right)}|X_{k-1}^{\left(i\right)}\right)=\frac{1}{{\left(2\pi \right)}^{\frac{15}{2}}{\left|Q_{k-1}\right|}^{\frac{1}{2}}}exp(-\frac{1}{2}|Q_{k-1}{|}^{-1}{\left({\hat{X}}_{k}^{\left(i\right)}\right)}^{2})$$

(34) $$q\left({\hat{X}}_{k}^{\left(i\right)}|X_{0:k-1}^{\left(i\right)},Z_{1:k}\right)=\frac{1}{{\left(2\pi \right)}^{\frac{15}{2}}{\left|{\hat{P}}_{k}^{\left(i\right)}\right|}^{\frac{1}{2}}}exp(-\frac{1}{2}|{\hat{P}}_{k}^{\left(i\right)}{|}^{-1}{\left({\hat{X}}_{k}^{\left(i\right)}-{\bar{X}}_{k}^{\left(i\right)}\right)}^{2})$$

## 4. Experiments and Results

### 4.1. Experimental Environment

**Figure 3:**
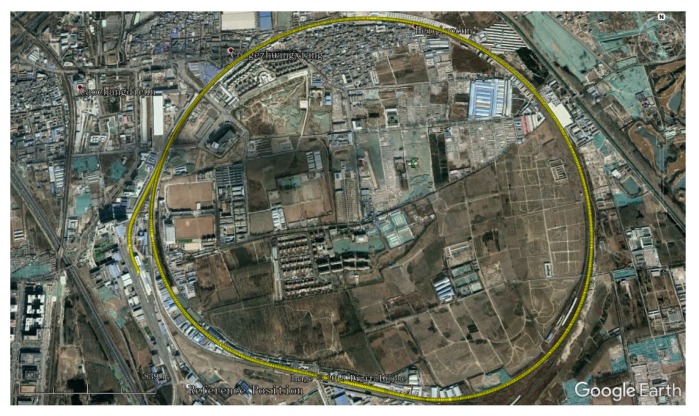
The Test Ring of the China Academy of Railway Sciences.

This section describes the test line selected for the on-site experiments. In order to verify the performance of the proposed adaptive algorithm, some tests were conducted on the Test Ring of the China Academy of Railway Sciences in Beijing. As shown in Figure 3, the test ring is around 9 km in length, and the speed of the train can be as high as 180 km/h.

**Figure 4:**
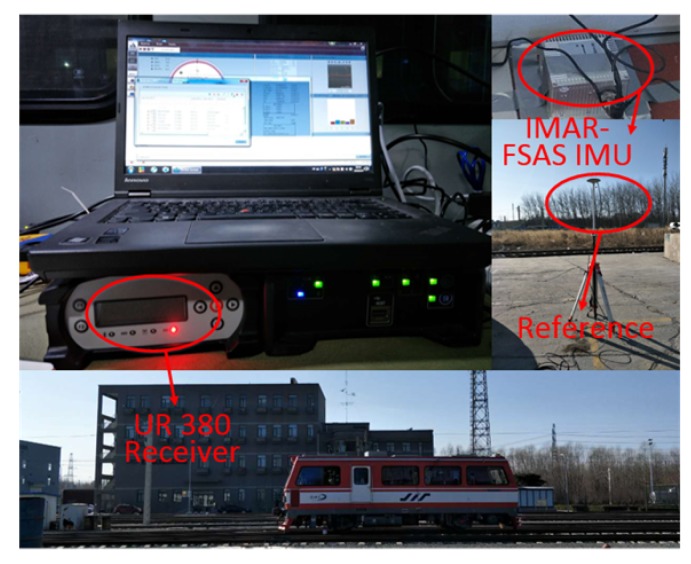
Onboard and trackside equipment.

The experimental equipment includes the trackside reference station and onboard system, which are illustrated in Figure 4. The IMAR-FSAS IMU records raw IMU data, and unicorecomm UR 380 multi-frequency GNSS receivers are adopted as the base station receiver, as well as the rover. The reference station is set in static mode, so that the RTK positioning solution can be obtained as the ground truth. Post-processing the rover data, accordingly, the DGNSS, PPP and standard point positioning (SPP) results are analysed to assess the proposed algorithm. As DTM plays a key role in it, in order to create an accurate digital track map, RTK results are interpolated to organize DTM, which consists of a series of point coordinates.

### 4.2. Results

**Figure 5:**
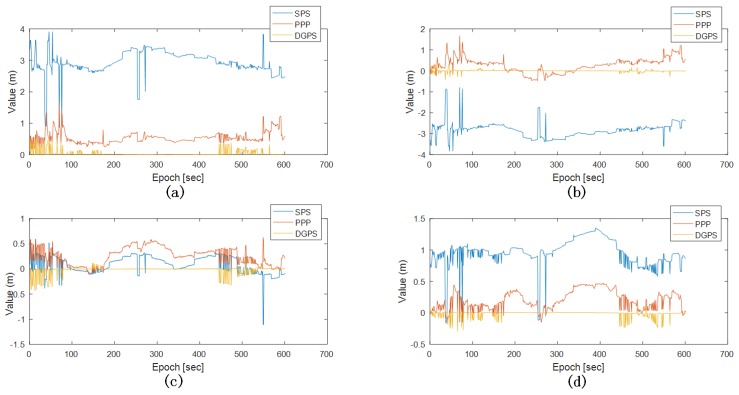
Positioning errors: (**a**) Positioning errors of SPP, PPP, DGPS; (**b**) Down errors of SPP, PPP, DGPS; (**c**) North errors of SPP, PPP, DGPS; (**d**) East errors of SPP, PPP, DGPS.

The test train was driven along the tracks with a maximum speed of 65 km/h; meanwhile, a deceleration and an acceleration in the range of 65 km/h to 0 km/h were included along the period of running. GNSS measurement output frequency was 1 Hz, Figure 5 reveals the SPP, PPP and DGPS positioning errors with respect to the RTK solution. It clearly shows that SPP had a meter-level positioning error, and PPP and DGPS obtained a decimetre-level one. DGPS had the best performance, especially when the test train was static during the operation.

**Figure 6:**
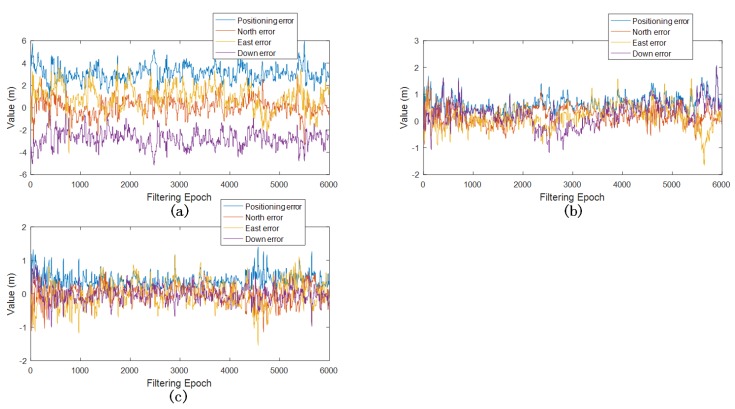
GNSS/INS EPF positioning errors with nominal variance: (**a**) SPP positioning errors; (**b**) PPP positioning errors; (**c**) DGPS positioning errors.

Without post-processing the results, usually prior GNSS measurement noise variances $\left(\sigma^{2}\right)$ were applied to model SPP, PPP and DGPS measurement noises, which are metre-level and decimetre-level, respectively. The positioning results of GNSS/INS EPF with nominal $\sigma^{2}$ (for SPP, PPP and DGPS, $\sigma^{2}$ are equal to 10, 1 and 1 respectively) are illustrated in Figure 6. It shows that the nominal variance values were feasible to be adopted in the filtering process. In general, these variance values are able to reflect positioning errors.

**Figure 7:**
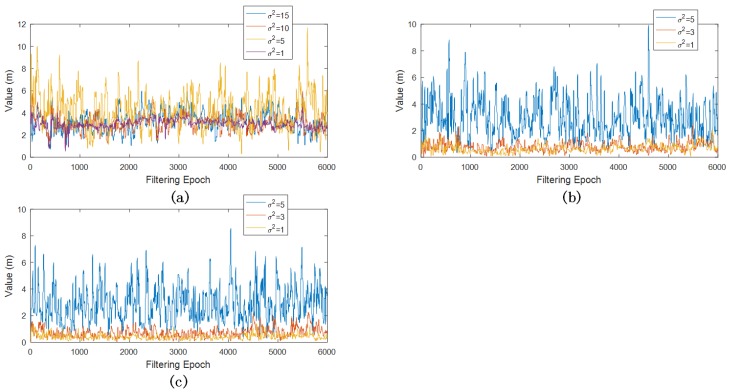
GNSS/INS EPF with different variances: (**a**) SPP positioning errors; (**b**) PPP positioning errors; (**c**) DGPS positioning errors.

Another fact we want to investigate is how different variance values will affect EPF results. Regarding SPP, typical positioning noise variance, values of 15, 10, 5 and 1 were fed into EPF, while 5, 3 and 1 were adopted for PPP and DGPS; thus, the filtering performance of SPP, PPP and DGPS with different $\left(\sigma^{2}\right)$ values, as shown in Figure 7, indicates that an exaggerated GNSS measurement noise covariance will considerably decrease positioning accuracy. On the other hand, a bold GNSS measurement noise variance (shown in SPP positioning errors in Figure 7) is acceptable for positioning accuracy, yet it damages the credibility of the positioning results, because the variance was overconfident. Therefore, it is important to adopt proper measurement noise variance values.

**Figure 8:**
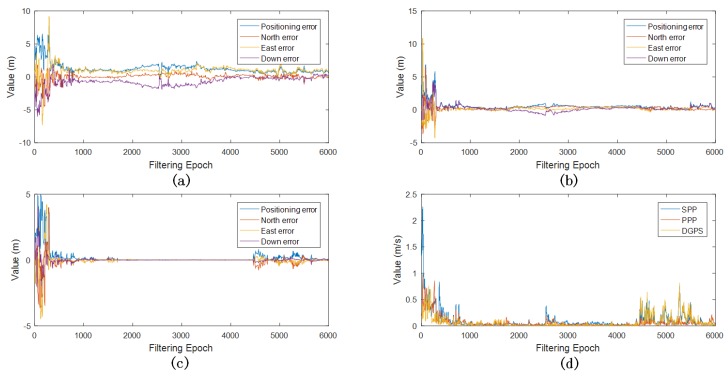
Adaptive filtering results: (**a**) SPP adaptive filter positioning errors; (**b**) PPP adaptive filter positioning errors; (**c**) DGPS adaptive filter positioning errors; (**d**) adaptive filter velocity errors.

**Figure 9:**
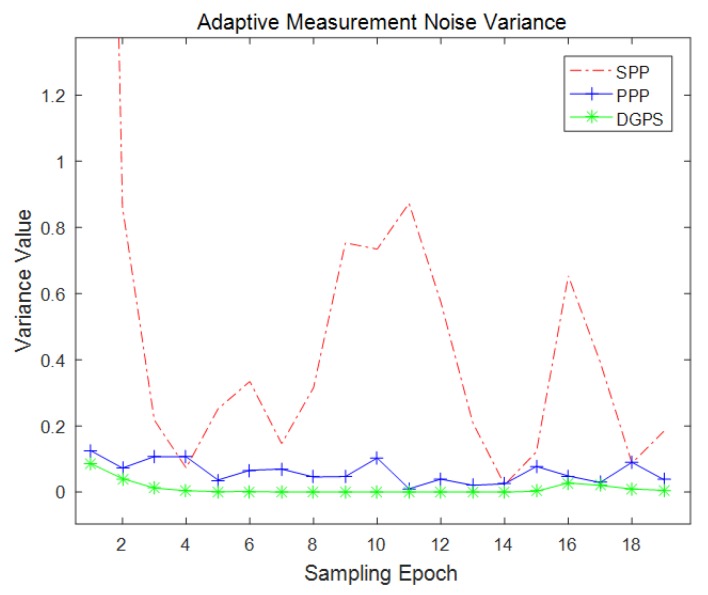
Adaptive GNSS measurement noise variance.

If the filter is able to estimate GNSS measurement noise variance itself, apparently the filter has the potential of performing better with respect to the accuracy of positioning. The proposed filter is the one estimating GNSS measurement noise variance based on extra DTM information. In order to implement GNSS measurement error sampling, a sampling period of 30 s was adopted, due to the fact that the stability of a small sample size will be affected by random gross error; on the contrary, a large sample size means more sampling time and less real time performance. The positioning results of proposed adaptive algorithm are revealed in Figure 8. In these tests, the algorithm set a prior GNSS measurement noise variance $\sigma^{2}=75$, which means the sole direction measurement noise standard deviation was five. The number of particle was 20,000, and the filtering period was 1 s, while the INS output frequency was 10 Hz. In order to further take advantage of DTM, the GNSS down-direction offset was estimated along with every sampling period, the GNSS velocity noise variance was set as one tenth of the position noise variance. Figure 8 shows that the filtering results converged after a few periods of sampling. The DGPS adaptive filter performed best when the test train was static in the middle of the test period. Meanwhile, the SPP and PPP adaptive filters showed some turbulence. Figure 9 describes the estimated GNSS measurement noise variance during adaptive filtering. the PPP and DGPS filters provided more stable estimation than the SPP filter. It is widely known that GNSS down-direction error is usually larger than that of other directions, which makes SPP positioning error much larger than 1 m at most times; however, the SPP measurement noise variance shown in Figure 9 is even less than one, due to the GNSS down-direction offset being estimated and finely eliminated along with every sampling period. This resulted in a remarkable improvement in accuracy in SPP. All three filters nonetheless provided more accurate and stable positioning results.

Some details are shown in Table 1, Table 2 and Table 3. Adaptive EPF after convergence in the following tables denotes adaptive GNSS/INS EPF filtering results after three periods of sampling. The results in this section clearly illustrate that the adaptive EPF algorithm took advantage of extra information provided by DTM. The position error mean reduced 58.6%, 11.5% and 47.6% in the SPP-, PPP- and DGPS-based filters in comparison to nominal EPF results, respectively. More importantly, the proposed algorithm obtained 62.4% and 14.9% improvements in position error mean in contrast to stand-alone SPP and PPP positioning solutions, respectively, considering the fact that usually the fusion results were approximate to or worse than the GNSS measurements.

## 5. Conclusions

This paper presents the positioning performance of a DTM-aided adaptive extended particle filter in relation to Standard Point Positioning (SPP), Precise Point Positioning (PPP) and Differential GPS (DGPS) techniques. The proposed algorithm was designed to estimate GNSS measurement noise variance adaptively based on added digital track map information, which makes the filter able to access the actual GNSS measurement noise variance. The GNSS down-direction offset is also estimated along with every sampling period by making full use of DTM information. Some on-site experiments were conducted in a nearly real railway environment. The results revealed in this paper demonstrate that the proposed adaptive algorithm gas the ability to decrease positioning errors in the fusion process, due to the introduction of DTM. The best performance improvement is observed in SPP/INS EPF. The adaptive filters even perform better than the SPP and PPP positioning due to the variance estimation of the adaptive GNSS measurement noise and the correction of the GNSS down-direction offsets. This paper proves the key role of DTM in positioning. The track map is a requisite for navigation and localization in railways. A better way of taking advantage of it results in more accurate positioning solution. In order to improve robustness, a failure detection process may be added in the proposed algorithm in future research. It can be a Kalman Filter-based scheme, since the proposed algorithm adopts EKF to estimate the unknown state vector and update the measurement covariance matrix. Alternatively, taking advantage of the particle filter, the state vector noise can be modelled as a non-Gaussian distribution.

## Figures and Tables

**Table 1:** Summary of the SPP positioning results.

	Stand-Alone	Nominal EPF	Adaptive EPF	Adaptive EPF after Convergence
Position error mean (m)	2.938	3.083	1.275	1.104
Position error variance	0.132	0.506	0.724	0.169

**Table 2:** Summary of the PPP positioning results.

	Stand-Alone	Nominal EPF	Adaptive EPF	Adaptive EPF after Convergence
Position error mean (m)	0.537	0.653	0.578	0.457
Position error variance	0.064	0.084	0.389	0.034

**Table 3:** Summary of the DGPS positioning results.

	Stand-Alone	Nominal EPF	Adaptive EPF	Adaptive EPF after Convergence
Position error mean (m)	0.045	0.414	0.217	0.074
Position error variance	0.015	0.040	0.449	0.014
